# Exploiting the features of deep residual network with SVM classifier for human posture recognition

**DOI:** 10.1371/journal.pone.0314959

**Published:** 2024-12-05

**Authors:** Irfan Kareem, Syed Farooq Ali, Muhammad Bilal, Muhammad Shehzad Hanif

**Affiliations:** 1 Department of Mathematics and Computer Science, University of Calabria, Rende, Italy; 2 School of Systems and Technology, University of Management and Technology, Lahore, Pakistan; 3 Department of Electrical and Computer Engineering, King Abdulaziz University, Jeddah, Saudi Arabia; 4 Center of Excellence in Intelligent Engineering Systems, King Abdulaziz University, Jeddah, Saudi Arabia; Najran University College of Computer Science and Information Systems, SAUDI ARABIA

## Abstract

Over the last decade, there have been a lot of advances in the area of human posture recognition. Among multiple approaches proposed to solve this problem, those based on deep learning have shown promising results. Taking another step in this direction, this paper analyzes the performance of deep learning-based hybrid architecture for fall detection, In this regard, the fusion of the residual network (ResNet-50) deep features with support vector machine (SVM) at the classification layer has been considered. The proposed approach outperforms the existing methods yielding an accuracy of 98.82%, 97.95%, and 99.98% on three datasets i.e. Multi-Camera Fall (MCF) using four postures, UR Fall detection (URFD) using four postures, and UP-Fall detection (UPFD) using four postures respectively. It is important to mention that the existing methods achieve accuracies of 97.9%, 97.33%, and 95.64% on the MCF, URDF and UPFD datasets, respectively. Moreover, we achieved 100% accuracy on the UPFD two-posture task. The URFD and MCF datasets have been utilized to assess the fall detection performance of our method under a realistic environment (e.g. camouflage, occlusion, and variation in lighting conditions due to day/night lighting variation). For comparison purposes, we have also performed experiments using six state-of-the-art deep learning networks, namely; ResNet-50, ResNet-101, VGG-19, InceptionV3, MobileNet, and Xception. The results demonstrate that the proposed approach outperforms other network models both in terms of accuracy and time efficiency. We also compared the performance of SVM with Naive Bayes, Decision Tree, Random Forest, KNN, AdaBoost, and MLP used at the classifier layer and found that SVM outperforms or is on par with other classifiers.

## Introduction

Human posture recognition has gained significant popularity due to its applications in healthcare [1], human-computer interaction [2], robotics [3], animal observation [4], surveillance [5], home automation [6], faint detection [7], home rehabilitation [8], sports analytics [9], driver assistance in intelligent transportation systems [10], and awkward posture recognition [11]. Moreover, approaches based on computer vision are playing an important role in sports analysis, player’s action recognition, and human fall detection [12, 13]. In the same vein, a lot of work has been done on human activity recognition especially fall detection for elderly people [14]. Human falls are the third major cause of unintentional deaths [15] and the annual cost of falls (due to ensuing medical bills) is about 31 billion per year in the United States [16]. Falling incidents mostly involved men over 65 years of age [17] and the world population of elderly aged 65 years is increasing [18]. It is expected to increase from 901 million to 1.4 billion by 2030, and to 2.1 billion by 2050 [19].

Fear of falls also leads to depression, social isolation, helplessness, and limits activities even at home [20]. In this context, improved human posture recognition using deep learning can play an important role in saving lives and providing timely medical assistance.

The substantial rise in research in this field led to the development of various algorithms and techniques for human posture recognition. Broadly, these techniques can be divided into two categories; sensor-based and vision-based [21]. Sensor-based approaches can either be wearable or non-wearable. Wearable sensor-based approaches contain devices such as gyroscopes, accelerometers, emergency buttons, and smartwatches [22]. However, these solutions may prove ineffective for elderly care where subjects may forget to wear such devices due to cognitive impairments [23]. Many low-cost systems using physiological parameter monitoring [24], inertial sensors [25] and the non-wearable sensor-based framework (using sound, vibrations or pressure) [26] exist but unreliability, high false alarm rate and the sensitivity to background noise are main concerns [26–28].

Due to high unreliability of non-wearable sensor-based framework and limitations of wearable sensors such as strict positioning along with inconvenience especially for the seniors who may even forget to wear them made the ambient sensor a choice of interest [29]. With recent advancements in computer vision, ambient sensors have begun using video-based methods for detecting human falls [21]. These methods could be broadly classified into two categories, namely: rule-based and machine learning-based [30]. Different sensors like pyroelectric infrared sensor [31], piezoelectric sensor [32], radio-frequency identification (RFID) sensors [33] and Kinect RGB-D camera [34] are used as ambient sensors.

Video obtained by the monocular camera has been widely used for human fall detection problems [29, 35, 36]. The solutions include single camera-based detection [36] where the fall detection methods depend on the extraction of the subject silhouette [37] or trajectory of the moving subject [38]. On the other hand, solutions based on multiple cameras utilize multi-cue analysis from multiple views [39] or 3D reconstruction [40] to detect falls. These existing video-based methods need to extract the subject first which is inclined to be influenced by image noise and illumination variation.

Vision-based approaches analyze frames or video for human posture detection [41] and rely on machine learning and computer vision [42]. Vision-based recognition tasks are challenging due to environmental factors e.g. illumination, shadows, occlusion [43], camouflage, non-uniform lighting, pose changes, camera view dependency, and non-lambertian surfaces [44]. Despite these challenges, a lot of work has been done in human detection [45], multi-camera tracking [37], gender recognition [46], and action analysis [47]. Literature on posture estimation and fall detection include feature engineering-based machine learning approaches, e.g. histogram of oriented gradients (HoG) and local binary pattern (LBP) features [48], silhouette description analysis [49]. Recently, Convolutional Neural Network (CNN) with the optical flow of RGB frames for fall/no-fall detection [45], deep neural network (DNN) regressors for human pose estimation [50] and OpenPose based multi-person pose detection [51] have also been proposed. Such machine learning-based posture/action recognition techniques have found utility in sports [52, 53] and surveillance systems [54]. With recent advances in deep learning, deep networks such as residual networks (ResNets), SqueezeNet, AlexNet, DenseNet, Inception, and Visual Geometry Group (VGG) have been used for posture/action recognition [55, 56]. The latest research suggests that increasing network depth requires careful optimization through rigorous experimentation [57]. Thus, hybrid approaches where pre-trained networks are combined with conventional machine learning classifiers have been considered for optimal computational complexity and time efficiency.

In this regard, Espinosa et al. proposed a multi-camera vision-based approach on the UP-Fall Detection (UPFD) dataset using optical flow features with an accuracy of 95.64% [58, 59]. They demonstrated the superiority of ResNet-based deep features classified through an SVM classifier achieving 99.98% accuracy. However, the UPFD dataset lacks real-life scenarios such as varying lighting conditions (ranging from daylighting to late evening). Kwolek and Kepski developed UR-Fall Detection Dataset (URFD) with a wide range of light variance and camera noise. Therefore, we also reported the performance of our approach using the URFD dataset. Wang et al [60] achieved an accuracy of 97.33% on this dataset while our approach achieves an accuracy of 97.95%. Camouflage and occlusion are challenging problems for successful fall detection, but these two datasets do not contain such challenging scenarios. Therefore, we also evaluated our approach using the Multi-Camera Fall (MCF) dataset which contains scenarios of camouflaged and occluded events [61]. Fan et al. [62] reported 97.9% accuracy for this dataset while our proposed methodology performed better with 98.82% accuracy.

Our proposed hybrid DNN architecture combines a residual neural network (ResNet-50) for in-depth feature extraction and a support vector machine (SVM) for classification. We have chosen SVMs as they are known to have better generalization capability because they minimize structural risk instead of empirical risk and are prone to overfit. They are also effective when dealing with high-dimensional feature space. RGB frame features from three distinct datasets are derived through the average pooling layer, following a sequence of convolution, pooling, and batch normalization operations within the ResNet-50 residual network. The resulting feature vector map is utilized for both training and testing phases using SVM. Our experimental datasets include MCF, URFD, and UPFD datasets. For MCF and URFD, we employed four posture categories, while for the UPFD, the experimentation involved two and four postures.

The following contributions are made in this paper.

- Performance analysis of the hybrid approach to classify ResNet-50 based deep features using a conventional robust classifier i.e. SVM for human posture recognition.
- Demonstration of the superior performance of the proposed approach in classifying the pose categories in the MCF, URFD, and UPFD datasets over existing methods in terms of percentage accuracy.
- Empirical evidence of the superiority of ResNet-50-based approach over other established deep architectures, such as ResNet-101, VGG-19, Inception-v3, MobileNet, and Xception, in terms of percentage accuracy on the considered datasets.
- Experimental evidence of either better or at par performance in terms of execution time compared to existing deep architectures.

## Related work

The research papers related to human pose recognition have been broadly classified into conventional machine learning and deep learning-based approaches.

### Conventional machine learning-based methods

In 2018, Zerrouki et al. proposed a human action classification scheme relying on the change in body shapes [63]. Their approach divided the human body into five parts and computed area ratios used as an input to the AdaBoost classifier, achieving an accuracy of 96.56% and 93.91% on the ‘University of Rzeszow’, and ‘University of Malaga’ fall detection datasets using six categories (bending, squatting, lying, sitting, walking, or standing). Maity et al. implemented human action recognition from silhouette images, using spatio-temporal body parts movement and trajectory analysis, achieving an accuracy of 93.75% and 95.06% on MuHVAi and Weizmann datasets [64]. Zhao et al. implemented a user-adaptive algorithm based on multivariate Gaussian distribution, k-Means clustering, and local outlier factor to detect human activities with an accuracy of 97.66% [65]. Mohd et al. used features of human height, variations in velocity, and acceleration of the subject to detect human fall [66]. Similarly, SVM was used to classify different daily life activities including walking, running, sitting, lying, and falling from three different datasets achieving an accuracy of 90.91%, 96.67%, and 97.39% respectively [67]. Yu et al. proposed shape and location-based features using one-class SVM for human fall detection [68]. In a previous work, we proposed an approach for human fall detection based on multiple features e.g. motion vector, aspect ratio, fall angle, centroid acceleration, head position, and foreground extraction using the MCF dataset achieving an accuracy of 96.58% [69]. The Weiyao et al. framework identified human actions using multilevel frame selection sampling (MFSS), which captures three levels of temporal samples from depth images [70]. They used motion and static maps (MSM) to extract features, and a KELM classifier was used for classification. They measured the accuracy of the three publicly available datasets MSRAction3D, MSRGesture3D, and UTD-MHAD at 98.2%, 98.3%, and 88.7%.

Ma et al. used an extreme learning machine classifier to distinguish falls from daily actions achieving an accuracy of 86.8% on a self-generated dataset [71]. Bian et al. used key joint information of depth images and head joints for human fall detection achieving an accuracy of 97.9% on a self-generated dataset [72]. Stone et al. presented a two-stage fall detection framework using the Microsoft Kinect [34]. Akagunduz et al. proposed shape sequence descriptor Silhouette Orientation Volume for classifying falls achieving an accuracy of 91.89% and 100% on SDU-Fall and Weizmann action dataset [73]. Özdemir et al. analyzed the appropriate position for wearable sensors on human body parts [74]. Doukas et al. proposed a feature-based human fall detection method based on sound and video information [75]. Rogier et al. proposed the Gaussian mixture model for fall detection on video sequences [29]. Ni et al. proposed motion and shape-based features (motion vectors and region of interest) for human fall detection [76] and reported an accuracy of 98.76% on their self-generated RGB-D dataset. In another of our previous works, we presented a feature-based approach using the J48 algorithm for human fall detection with an accuracy of 99.2% using the MCF dataset [77]. Kasturi et al. used a top-view Kinect camera and proposed a framework based on the shape and orientation features for the classification of various human activities in the URFD dataset [78]. Recently, Merrouche et al. proposed an approach for elderly fall detection using shape deformation and motion information [79]. They used a histogram of distance orientation and center of mass velocity along with Adaboost and achieved an accuracy of 95.45% and 98.41% on the URFD and SDU Fall datasets respectively. Table 1 highlights the respective performances of various techniques on standard datasets based on the above discussion.

**Table 1:** Summary of related works based on conventional machine learning methods.

Ref.	Proposed	Result	Dataset
2018, Zerrouki et al. [63]	Human Body shapes, area ratios using AdaBoost Classifier	96.56% accuracy for ‘University of Rzeszo’, 93.91% accuracy for ‘University of Malaga’ fall detection datasets	University of Rzeszo, University of Malaga
2017, Maity et al. [64]	Spatio-temporal body parts movement and trajectory analysis from silhouette images	93.75% and 95.06% accuracy on MuHVAi and Weizmann datasets	MuHVAi, Weizmann
2013, Ali et al. [69]	Multiple features e.g. motion vector, aspect ratio, fall angle, centroid acceleration, head position, and foreground extraction based Human fall Detection	96.58% accuracy	MCF
2019, Weiyao et al. [70]	Motion and static maps (MSM) to extract feature with KELM classifier	Accuracy of 98.2%, 98.3%, and 88.7% on MSRAction3D, MSRGesture3D, and UTD-MHAD	MSRAction3D, MSRGesture3D, UTD-MHAD
2016, Akagunduz et al. [73]	Shape sequence descriptor Silhouette Orientation Volume for classifying falls	Accuracy of 91.89% and 100% on SDU-Fall and Weizmann action dataset	SDU-Fall, Weizmann action
2018, Ali et al. [77]	Feature-based approach using the J48 algorithm	99.2% accuracy	MCF
2017, Kasturi et al. [78]	Shape and orientation features for classification with Binary SVM	96.34% accuracy	URFD
2020, Merrouche et al. [79]	Shape deformation, motion information, histogram of distance orientation and center of mass velocity with Adaboost classifier	Accuracy of 95.45% and 98.41% on URFD and SDU Fall	URFD and SDU Fall

### Deep learning-based methods

In 2017, Li et al. used AlexNet for fall detection using the URFD dataset% [92]. Solbach et al. proposed a convolution neural network based on audio and visual cues and achieved an accuracy of 91% [93]. Ge et al. proposed a fall detection scheme based on VGG-16 features and sparse dictionary learning with SVM for classification, achieving an accuracy of 90.5% on the MCF dataset [94]. Han et al. used images from Microsoft Kinect and proposed deep neural networks for human posture recognition [95]. In 2018, Steven et al. used spectrogram-based approach and deep LSTM network to achieve an accuracy of 88.14% on the UCI dataset [80]. Elforaici et al. used transfer learning and joint configuration to model 3D postures [96]. Min et al. used faster R-CNN to detect daily life activities including, sitting, and lying down, and achieved 95.5% accuracy on the URFD dataset [81]. In their study, Taramasco et al. proposed a human fall detection system for elderly people using low-resolution thermal sensors in conjunction with three deep neural networks: long short-term memory (LSTM), gated recurrent unit, and Bi-LSTM [97]. Their monitoring system had 93% accuracy in detecting falls with Bi-LSTM. In a study by Zhang et al., they presented trajectory-weighted deep-convolutional rank-pooling descriptors (TDRDs) for human fall detection that works well in interfered environments [82]. With the proposed clustering and rank pooling technique, the TDRD has fewer redundant features. With SVM classifiers, an accuracy of 96.04% was achieved on the SDUFall dataset, while a sensitivity of 90.21% and 100% was achieved on the MCF and the URFD datasets, respectively.

In 2019, Khraief et al. introduced a VGGNet-16-based architecture that utilizes the human region, its histogram, and the orientation of optical flow as input, achieving a specificity of 92.5% on the URFD dataset [83]. Aubry et al. employed a deep residual network for human action recognition with the NTU RGB-D dataset [56]. Noori et al. utilized LSTM with the OpenPose library for human posture recognition, achieving an accuracy of 92.4% on the Berkley MHAD dataset [84]. In a more recent study, Khraief et al. employed VGG-16 for elderly fall detection based on motion and shape information [85]. They applied MCF and URFD datasets, achieving specificities of 99.80% and 95%, respectively. Liu et al. proposed an energy-guided temporal segmentation network using the NTU RGB-D dataset, achieving an accuracy of 94.7% [98]. Han et al. presented a two-stream method for fall detection using MobileVGG, considering motion parameters of the human blob and lightweight VGG networks [86]. They achieved 98.2% accuracy on a self-collected dataset through 200 epochs of training and Adam’s optimizer.

In 2020, Wang et al. proposed dual-channel feature integration for elderly fall detection [60]. They employed Yolo and OpenPose for object detection and posture recognition, achieving accuracies of 97.33% and 96.91% on the URFD and Le2i fall detection datasets. Asif et al. suggested a deep architecture, FallNet, for human fall detection [87], achieving F1 scores of 0.986 on the MCF dataset and 0.735 on the Le2i Fall dataset. In a recent work, we proposed an optimized residual network architecture for elderly fall detection using the OpenPose library for human skeleton detection [88], obtaining an accuracy of 97.46% on camera 2 videos of the MCF dataset.

Kulikajevas et al. [99] introduced an extension to the MobileNetV2 neural network, incorporating hierarchical data representation. This extension enables the deep neural network to extract crucial temporal information from video frames, utilizing sequential video data as input. The proposed posture classification technique, characterized by its flattened tree representation, exhibits extensibility and seamless applicability to contemporary posture classification tasks. The depth of the ontological semantic posture model serves as a pivotal variable influencing classification quality. For predicting three primary sitting posture classes (straight posture, forward posture, and backward posture), the suggested technique achieves a classification accuracy of 91.47%.

Liu et al. concluded that domain-adaptive fall detection (DAFD) proves to be an efficient approach for addressing cross-domain challenges, such as cross-position and cross-configure problems, through the application of deep adversarial training (DAT) [100]. Utilizing DAFD on the UPFD and UMA Fall datasets, the proposed method minimizes domain discrepancies to mitigate mismatch issues, enhancing detection performance by 1.5 to 14% compared to typical fall detection systems. In a comprehensive review by Khan et al., fall detection techniques were categorized based on the availability of sufficient fall training data and the absence or insufficiency of such data [101]. The proposed taxonomy, independent of sensor types and specific feature extraction/selection methods, classifies various fall classification methods. Neili et al. introduced a technique for human posture recognition using skeleton data from Kinect [102]. Employing CNN and multiclass support vector machines, the method predicts joint positions and identifies posture classes, achieving a precision of 98.07% on the Cornell Activity Dataset (CAD60). Chen et al. presented a video-based fall detection method utilizing a lightweight pose estimator to extract 2D poses from video sequences [103]. These 2D poses are then transformed into 3D poses to recognize fall events based on the estimated 3D poses. On the NTU RGB+D benchmark action recognition dataset, the method achieves an accuracy of 99.83%. Most recently, Inturi et al. proposed a convolutional neural network with long short-term memory (CNN+LSTM) architecture and analyzed the human fall detection by applying AlphaPose pre-trained network to acquire the human body prime joint points [89]. On the UPFD dataset, they achieved an accuracy of 98.59% on different falls and daily life activities. Mobsite et al. introduced a surveillance camera-based framework for fall detection [90]. They utilized the Multi-Scale Skip Connection Segmentation Network (MSSkip) to extract human silhouettes from raw images. Additionally, Convolutional Long Short-Term Memory (ConvLSTM) was employed to detect human poses. Their method achieved an F1-score of 97.68% and 97.58% on the UPFD and URFD datasets respectively. With the Xception network, they achieved an F1-score of 97.85% on the customized post-fall dataset. Su et al. devised a hybrid approach merging lightweight 3D-CNN and ConvLSTM networks to augment the discriminative features with channel- and spatial-wise attention modules, and leveraged ConvLSTM for long-term spatial-temporal feature extraction [91]. Their approach achieved an accuracy of 62.55%, 97.28%, 98.06%, and 94.84% on the HMDB5, UCF11, URFD, and MCF datasets respectively. The diverse techniques, along with their respective performances on standard datasets, are summarized in Table 2.

**Table 2:** Summary of related works based on deep learning methods.

Ref.	Proposed	Result	Dataset
2017, Li et al. [79]	Modify AlexNet for fall detection	Accuracy of 99.98%	URFD
2018, Steven et al. [80]	LSTM network	Accuracy of 88.14%	UCI
2018, Min et al. [81]	faster R-CNN to detect daily life activities	Accuracy of 95.5%	URFD
2018, Zhang et al. [82]	Trajectory-weighted deep-convolutional rank-pooling descriptors (TDRDs), clustering and rank pooling technique with SVM classifiers	Accuracy of 96.04% on SDUFall dataset, 90.21% and 100% sensitivity on MCF and URFD datasets	SDUFall, MCF, URFD
2019, Khraief et al. [83]	Human region histogram, and orientation of optical flow with VGGNet-16	Specificity of 92.5%	NTU RGB-D
2019, Aubry et al. [56]	RGB-D videos, Human skeleton detection with Openpose with Deep residual network	Accuracy of 83.317% cross-subject and 88.780% cross-view	NTU RGB+D
2019, Noori et al. [84]	LSTM with OpenPose library	Accuracy of 92.4%	Berkley MHAD
2020, Khraief et al. [85]	VGG-16, fall detection based on motion and shape information	specificity of 99.80% and 95% MCF and URFD datasets	MCF, URFD
2020, Han et al. [86]	Two-stream method of fall detection using the MobileVGG, considers motion parameters of the human blob	Accuracy of 98.2%	Self-collected
2020, Wang et al. [60]	Yolo and OpenPose for object detection and posture recognition	Accuracy of 97.33% and 96.91% on URFD and Le2i fall detection datasets	URFD, Le2i fall detection
2020, Asif et al. [87]	Deep architecture FallNetfor human fall detection	F1 scores of 0.986 on the MCF dataset and 0.735 on the Le2i Fall dataset	MCF, Le2i fall detection
2020, Kareem et al. [88]	Optimized residual network architecture with Openpose library for human skeleton detection for Human Fall detection	Accuracy of 97.46%	MCF (Camera 2)
2023, Inturi et al. [89]	CNN + LSTM	Accuracy of 98.59%	UPFD
2023, Mobsite et al. [90]	ConvLSTM and Xception network	F1-score of 97.68% and 97.58%	UPFD and URFD
2024, Su et al. [91]	3D-CNN and ConvLSTM	Accuracy of 62.55%, 97.28%, 98.06% and 94.84%	HMDB5, UCF11, URFD, and MCF

## Methodology

In the traditional classification scheme, hand-crafted features are fed to a classifier for the task at hand. These features require feature engineering and domain experts. Traditional methods may also struggle with capturing complex patterns in data, especially in high-dimensional spaces or with unstructured data like images and text. At present, several well-known pretrained CNN architectures are available for general-purpose classification tasks. Deep convolutional networks, like ResNet-50, excel at learning and capturing intricate patterns and structures from raw data. However, they require large amounts of data to generalize well and avoid overfitting. Pre-trained networks trained on large-scale datasets like ImageNet are therefore employed to extract features directly from the image which are not only discriminative but also robust and are used for classification tasks in computer vision. These features are even beneficial in scenarios that are not seen by the pre-trained network. However, if the new task has a different data distribution that does not match with the train set distribution, a fine-tuning approach is employed where the final classification layer (a fully connected layer) or the whole network is adapted by training it with the data of the new task. For this reason, we have considered ResNet-50 as the base architecture for deep feature extraction. Moreover, ResNet-50 strikes a balance between depth and computational complexity since it is deeper than VGG-19 but less complex than ResNet-101. Deeper networks can capture more intricate features, but they may suffer from vanishing gradients during fine-tuning if needed. ResNet-50 mitigates this issue with residual connections. Furthermore, ResNet-50 has achieved remarkable performance on the ImageNet dataset. The experimental data provided later indicates that indeed the choice of ResNet-50 against other architectures such as ResNet-101, VGG-19, MobileNet, InceptionV3, and Xception etc. for the task of human pose estimation yields promising results.

One critical choice is the final classifier, and it affects the overall performance of the network. Due to the high dimensionality of the feature vector extracted by the deep network before the classification layer, a strong regularization is needed to train or fine-tune the network in an end-to-end manner. For this purpose, a regularization scheme like dropout is used. However, the use of a dropout layer increases the overall training time as only a portion of weights depending on the dropout factor gets updated at every training step. On the other hand, existing statistical machine learning algorithms like SVMs provide an alternative solution. SVMs are known to have better generalization capability because they minimize structural risk instead of empirical risk. Moreover, they are prone to overfit and effective when dealing with high-dimensional feature space and smaller datasets. Additionally, they are also computationally efficient once the feature vectors are available.

**Fig 1:**
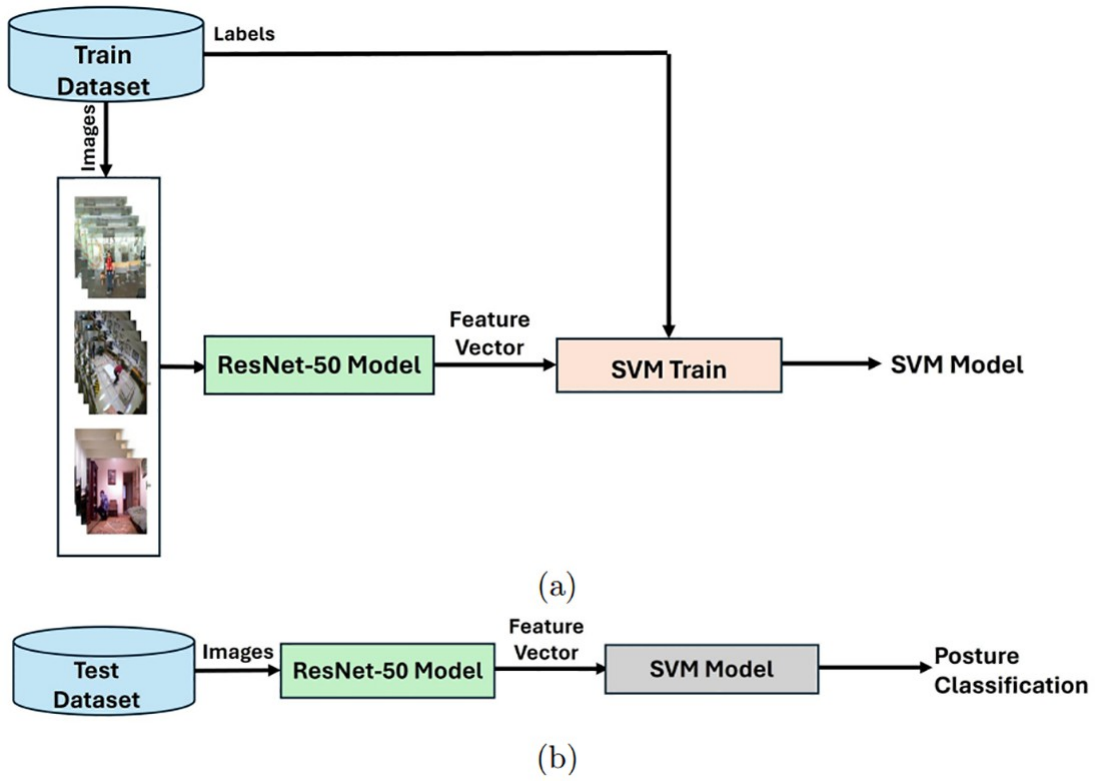
Proposed framework: (a) Training of an SVM classifier using ResNet-50 features, (b) Posture classification using the trained SVM model and ResNet-50 features.

Thus, this study proposes a hybrid detector that utilizes the discriminative power of the ResNet-50 deep neural network for human posture recognition problem through an SVM classifier. Fig 1 demonstrates the training and test stages in our proposed framework. During the training stage, an SVM model is trained using the ResNet-50 features obtained from the average pooling layer of the network by doing a forward pass on the train images. Associated labels are also fed to the training algorithm of the SVM model. We used a pre-trained ResNet-50 model which was trained on the ImageNet image classification dataset. As the ResNet-50 has seen millions of images during training, the extracted features are usually considered as useful and suitable for other computer vision tasks. The test stage is composed of extracting ResNet-50 features for input images followed by the trained SVM model to classify them into posture categories.

**Fig 2:**
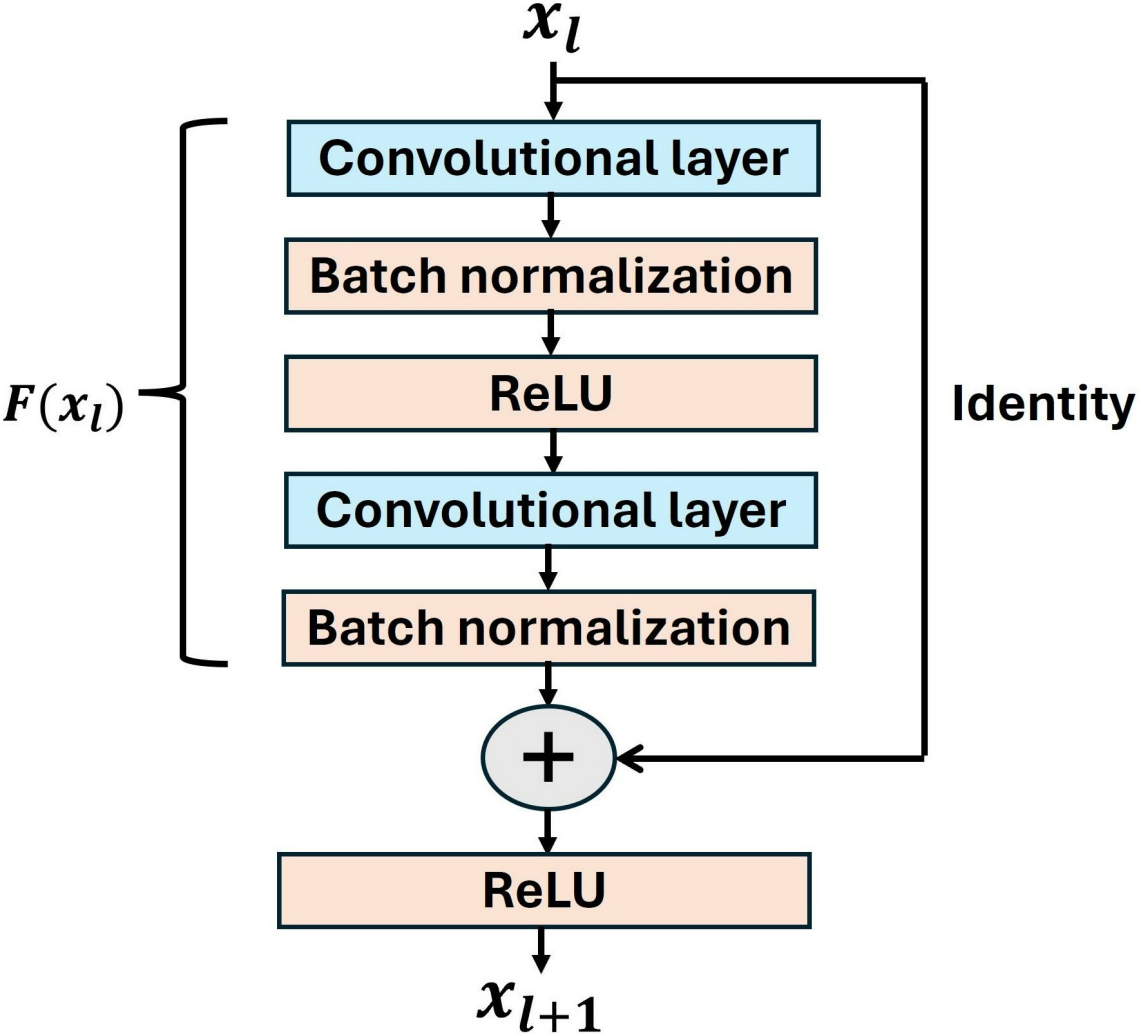
A residual block.

Residual networks [55] outperform plain convolutional neural networks due to their unique architecture. They are composed of several residual blocks where each block consists of a stack of convolutional (conv), batch normalization (bn), Rectified Linear Unit (ReLU) layers and a skip connection from input to output to perform an identity mapping. A typical residual block is shown in Fig 2. The conceptualization of residual networks is based on the fact that very deep plain networks do not perform well as the gradient of the loss function becomes too small during backpropagation [104] and eventually vanishes yielding degraded performance. The unique architecture of residual networks addresses this issue of vanishing gradients. A residual block uses a skip connection from the input to output serving as an identity mapping function. The residual branch is composed of conv-bn-ReLU-conv-bn layers to learn the underlying mapping *F*(*x*_*l*_) for the specific task for which the network is being trained. We can write the output *x*_*l*+1_ of *l*^*th*^ residual block for an input *x*_*l*_ as:

$$\begin{array}{c} x_{l+1}=ReLU\left(F\left(x_{l}\right)+x_{l}\right) \end{array}$$ (1)

For classification purposes, the features are extracted from the global average pooling (GAP) layer of the ResNet-50 network. The GAP has the advantage over fully connected dense layers as it enforces correspondence between feature maps of different categories due to its native convolution structure [105]. The GAP does not require optimization due to its parameter limitation. Besides, it is robust to the spatial translations as it sums up the spatial information. After a series of convolution operations in the residual block, the input size of 128 × 128 × 3 was changed to 4 × 4 × 2048. Later, the GAP layer provides a 2048 dimensional feature vector. The dataset is split into 9:1 as training and validation samples for the SVM training.

Suykens et al. proposed SVM, an optimal margin separating classifier that promises high generalization ability [106, 107] and handles data non-linearity intrinsically in its dual formation using the kernel trick. To find the optimal hyperplane of the n-dimensional feature map, SVM uses regularization parameters and kernel/loss function as described below in 2 [106, 107].

$$\begin{array}{c} \underset{\omega ,\xi }{\text{min}}\;\frac{1}{2}\omega^{T}\omega +C\sum_{i=1}^{N}\xi_{i} \end{array}$$ (2)

where *C* is the regularization parameter, *ξ* illustrates slack variables, and *ω* is the weight vector.

## Experiments & results

In this section, we present our experimental setup including the description of the used datasets and the experimental results. This includes comparison of the proposed approach (denoted as PA) with six networks including ResNet-50, ResNet-101, VGG-19, MobileNet, InceptionV3, and Xception for a four-category classification problem of human posture recognition. These four categories included: standing and walking, lying on the ground, moving down or falling, and moving up or jumping. This study also discusses the two-category problem (Fall/No-Fall) using the UPFD dataset containing videos with five different types of falls. We measured the classification accuracy and execution time of our proposed approach on three publicly available datasets: MCF, URFD, and UPFD using 20 epochs. The results provided in this section provide the empirical evidence behind the decision to choose ResNet-50 as the base architecture in the proposed approach for extracting deep features in the context of human pose estimation.

### Environment setup

Experiments were executed on the Google Colab utilizing the GPU-based hardware acceleration using a single NVIDIA Tesla P100 GPU with 16 GB memory. Proposed and state-of-the-art benchmark architectures were implemented on the same datasets. The base models were imported using Keras. Due to the large number of frames in the UPFD dataset, the 16 GB memory was insufficient. Hence, an extended RAM option of 25/35 GB with GPU/TPU was used for the corpus size of more than 35k frames.

### Dataset

We have used three vision-based datasets in our experiments the details of which have been provided in the following subsections.

#### Multi-Camera Fall (MCF) dataset

**Fig 3:**
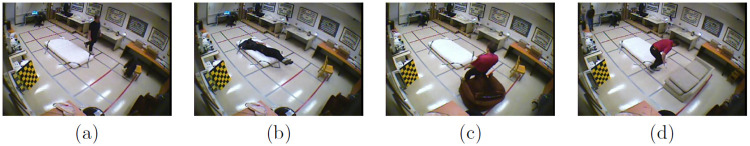
Key frames of MCF dataset showing different postures: (a) Standing or walking, (b) Lying on ground, (c) Moving down or sitting, (d) Standing up.

We utilized the MCF dataset [61], comprising 24 videos captured by eight affordable IP cameras denoted as Camera 1 (C1), Camera 2 (C2), Camera 3 (C3), Camera 4 (C4), Camera 5 (C5), Camera 6 (C6), Camera 7 (C7), Camera 8 (C8). Consequently, the dataset encompasses 192 videos exhibiting diverse human postures, encompassing both routine activities and instances of human falls. Daily life activities encompass sitting down, standing up, housekeeping, walking around the mattress, and crouching down, while human falls include backward falls, forward falls, sitting down, and falls due to loss of balance. Fig 3 illustrates keyframes depicting a range of human postures, including walking and standing, falling/lying on the ground, moving down, and moving up.

The experiments conducted in this study used 22 videos comprising four postures i.e., walking or standing, fall/lying on the ground, moving down, and moving up. Frames were extracted from videos of all eight cameras. The statistics of the frames corresponding to these videos are given in Table 3. It is to be mentioned that as all eight cameras are synchronized, the number of frames for each posture is the same across all cameras.

**Table 3:** Number of frames for each posture of four datasets namely MCF, URFD, UPFD-Four Postures (UPFD-4P), and UPFD-Two Postures (UPFD-2P).

Dataset	P1	P2	P3	P4	Total
MCF-C1 to C8	4189	5513	2783	1462	13947
URFD	4243	5567	2837	1516	14163
UPFD-4P	18247	20710	2780	8903	50640
UPFD-2P	18132	15382	-	-	33514

Posture P1 corresponds to walking and standing, whereas P2, P3 and P4 represent, fall/lying on the ground, moving down, and moving up respectively.

#### UR-Fall Detection (URFD) dataset

**Fig 4:**
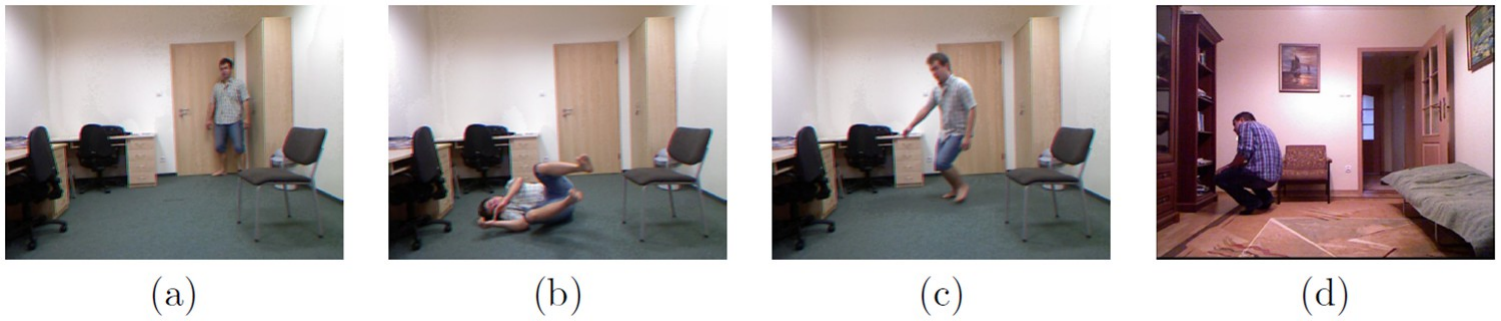
Key frames of UR Fall dataset with varying postures: (a) standing or walking, (b) lying on ground, (c) moving down or falling, (d) standing up.

We used the URFD dataset (available at http://fenix.univ.rzeszow.pl/mkepski/ds/uf.html) developed by Kwolek and Kepski [108] that contained 70 activities from 5 subjects, comprising 30 falls and 40 daily life activities. Two sensors, one positioned at a height of one meter and the other at the ceiling, were used for recording the fall events. The Kinect sensor simultaneously captured depth and color images at a frame rate of 30 fps. Fig 4 shows the keyframes corresponding to various postures. We used 30 Fall and 40 ADL sequences of Camera 0 in our experiments. The statistics of RGB frames corresponding to various postures including walking, fall, moving down, and moving up are shown in Table 3.

#### UP-Fall detection (UPFD) dataset

**Fig 5:**
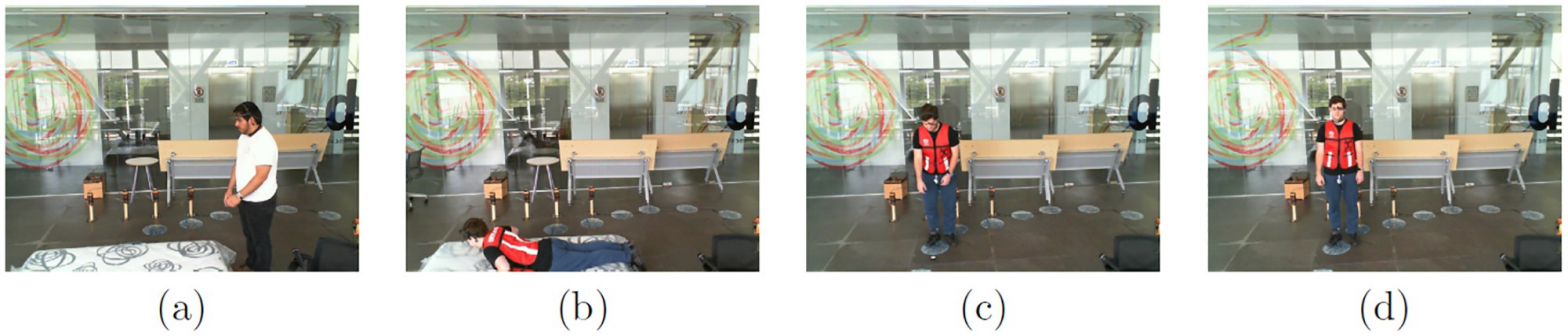
Key frames of UPFD dataset with varying postures: (a) standing or walking, (b) lying on the ground, (c) moving down or falling, (d) standing up or jumping.

We used the UPFD dataset (available at https://sites.google.com/up.edu.mx/har-up/) developed by Martínez et al. [109] that consisted of 11 activities performed by 17 young people. We used the lateral view of the camera to capture four postures: walking and standing, fall/lying on the ground, moving down or falling, and standing up or jumping. These postures are generated in three trials using 17 subjects. Moreover, five types of falls are also generated including forwarding fall, backward fall, sideward fall, and fall using knees and hands. Fig 5 illustrates some of the keyframes corresponding to various postures.

### Experimental results on the MCF dataset

Our proposed network architecture outperforms the existing state-of-the-art architectures on all but two of the videos of the MCF dataset as shown in Table 4. The proposed approach uses SVM as a classification layer and achieves an accuracy of 98.82% as compared to 96.96% obtained using simple ResNet-50 whereas, in terms of time efficiency, its results are comparable to the simple ResNet. It was empirically observed that the combination of deep residual network features and SVM classifier was helpful for datasets containing high intra-class variations such as MCF. The reason for the high variations in the MCF dataset was the data from eight cameras placed at different locations and orientations. Auvinet et al. described the placement of 8 IP Cameras (C1 to C8) in the room [61]. Other factors like a shadow, reflection due to light effects, cluttered and composed background, different cloth colors, and variable illuminations also added to the complexity. It can be observed from Table 5 that the proposed approach is 1.5 times faster than ResNet-101 (which has a higher number of layers to train) and takes slightly more time than VGG-19, InceptionV3, MobileNet, and Xception (with fewer number of layers). Results are reported based on RGB frame inputs. Table 6 shows that the proposed approach outperforms other relevant works reported in the literature in terms of percentage accuracy.

**Table 4:** Comparison of the proposed approach (PA) in terms of accuracy (%) with existing architectures on the MCF dataset.

Camera No.	R-50	R-101	V-19	I-V3	M-Nt	Xp	PA
C1	96.54	79.60	40.35	96.69	97.35	97.79	**99.18**
C2	97.82	91.94	40.05	98.80	95.42	99.13	**99.20**
C3	98.67	98.33	40.81	98.68	98.59	**98.93**	98.37
C4	**98**.11	95.76	39.17	94.56	97.17	97.02	97.54
C5	98.01	95.72	41.37	97.79	91.89	98.23	**99.18**
C6	92.53	97.73	40.00	89.38	94.36	97.86	**99.20**
C7	97.88	97.73	38.30	96.13	97.96	98.39	**98.83**
C8	96.15	86.46	40.09	90.61	95.04	94.82	**99.11**
Average	96.96	92.90	40.01	95.33	95.97	97.77	**98.82**

R-50 = ResNet-50, R-101 = ResNet-101, V-19 = VGG-19, I-V3 = InceptionV3, M-Nt = MobileNet, Xp = Xception.

**Table 5:** Comparison of proposed approach (PA) in terms of time (sec) with existing architectures on MCF dataset.

Camera No.	R-50	R-101	V-19	I-V3	M-Nt	Xp	PA
C1	4380	7480	2680	3000	1520	4740	**4627**
C2	2820	3000	780	1300	700	1540	**3256**
C3	2780	4380	1800	1740	1300	2420	**3040**
C4	1820	2860	780	1700	1280	3340	**2018**
C5	4340	7540	2620	1760	1300	4620	**4516**
C6	4320	7420	2680	2980	2980	2420	**4474**
C7	4340	7480	2680	2980	1500	4700	**4546**
C8	4160	7280	2500	2920	1440	4420	**4356**
Average	3620	5930	2065	2298	1503	3525	**3854**

R-50 = ResNet-50, R-101 = ResNet-101, V-19 = VGG-19, I-V3 = InceptionV3, M-Nt = MobileNet, Xp = Xception.

**Table 6:** Comparison of existing approaches with proposed approach (PA) in terms of accuracy (%) on MCF dataset.

Dataset	KFan [110]	Lu [111]	Fan [62]	Ge [94]	Si [112]	Wa [54]	PA
MCF	94	97.27	97.9	90.5	95.6	90.3	**98.82**

### Experimental results on the URFD dataset

**Fig 6:**
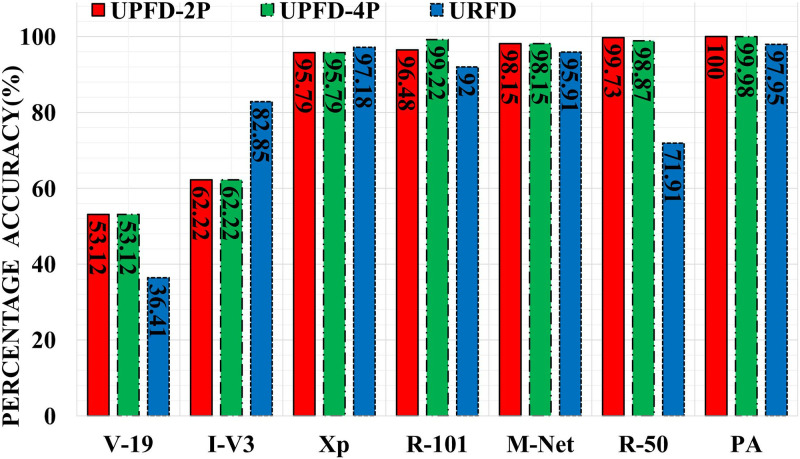
Comparison of the proposed approach (PA) in terms of accuracy (%) with existing architectures on URFD dataset and UP-Fall detection dataset.

**Fig 7:**
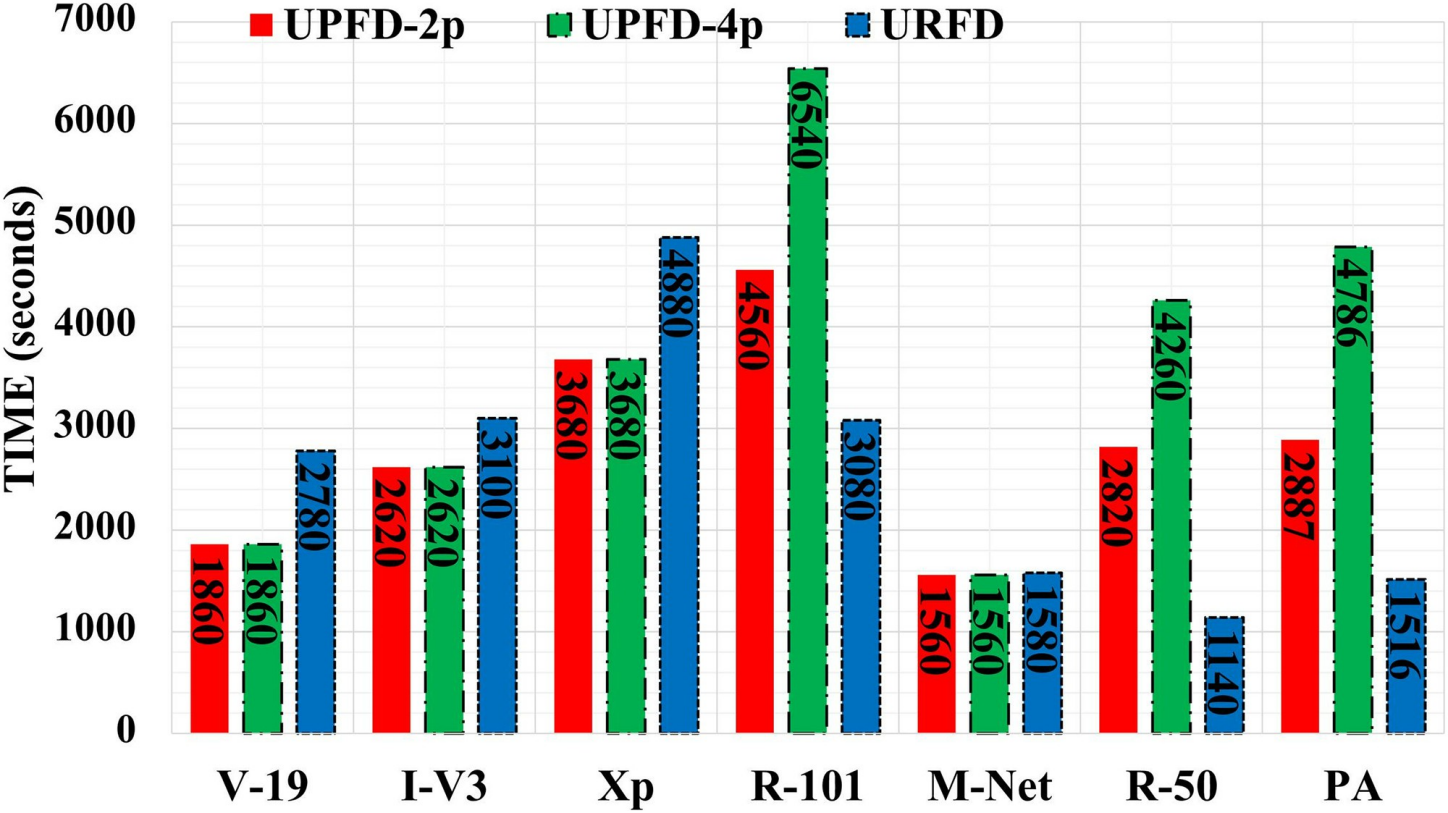
Comparison of proposed approach (PA) in terms of time (sec) with existing architectures on URFD dataset and UP-Fall detection dataset.

When using the URFD dataset, the proposed approach outperformed the existing deep architectures in terms of accuracy (Fig 6). ResNet-50 and InceptionV3 degraded in percentage accuracy by 25.84%, and 13.09% respectively as compared to the MCF dataset, due to the dim light conditions and lack of clarity in the URFD dataset (especially in the initial frames). However, the proposed approach remained stable on the URFD dataset due to the robustness of ResNet-50 features and the optimal margin separating SVM classifier [113]. The accuracy of VGG-19 remained low as it needed a longer time to converge and required a large training data size [114]. The proposed approach is {2.03, 1.83, 2.04, 1.04, 3.22} times faster than ResNet-101, VGG-19, InceptionV3, MobileNet, and Xception respectively as shown in Fig 7. The percentage decrease in execution time (training and testing time) of the proposed approach is 59.6% on the URFD dataset, as compared to the MCF dataset, due to the learning complexity in the MCF dataset (shadow, cluttered and composed background, variable illuminations, and reflection due to light effects). Moreover, Table 7 shows that the proposed approach outperforms the competition in terms of accuracy on the URFD dataset for human posture recognition.

**Table 7:** Comparison of existing approaches with proposed approach (PA) in terms of % accuracy on URFD dataset.

Dataset	Kh [85]	Wa [60]	Kh [83]	Mi [81]	Ge [94]	Mo [66]	PA
URFD	95	97.33	92.5	95.5	84	96.67	**97.95**

### Experimental results on the UP-Fall dataset

The proposed approach is compared with existing architectures using two and four postures of this dataset which contains varying lighting conditions, shadows, and different clothing of the subjects.

#### With two postures

Our proposed architecture outperforms ResNet-50, ResNet-101, VGG-19, MobileNet, InceptionV3, and Xception using two postures (‘Fall’&‘No Fall’) as shown in Fig 6. ResNet-101, VGG-19, and MobileNet exhibited a percentage increase of 3.58, 13.11, and 2.18 respectively on this dataset as compared to the MCF dataset. If compared with the URFD, ResNet-50 exhibited the highest percentage increase of 25.05 while the second-highest percentage increase of 12.48 is shown by InceptionV3. The reason for good performance is the larger training data size. Moreover, this dataset has low intra-class variations as compared to MCF and URFD. As can be seen in Fig 7, the proposed approach is 1.58 and 1.27 times faster than ResNet-101 and Xception.

#### With four postures

The proposed approach outperforms ResNet-50, ResNet-101, VGG-19, MobileNet, InceptionV3, and Xception on the UPFD dataset with four varying postures (see Fig 6) and achieves the highest classification accuracy of 99.98% while maintaining a comparable running time. The proposed approach is 1.37 times faster than ResNet-101 (see Fig 7). The proposed and existing architectures take more time in classifying this dataset as compared to MCF and URFD, because of the larger number of frames.

The proposed approach outperforms other approaches in terms of percentage accuracy on this dataset for human posture recognition as can be seen in Table 8. The combination of ResNet features and SVM classifiers in the proposed approach works well in a dataset with low intra-class variations.

**Table 8:** Comparison of existing approaches with proposed approach (PA) in terms of accuracy (%) on UPFD dataset.

Dataset	Co [115]	EsPo [58]	Es [59]	Ri [116]	Ma [117]	MaPo [109]	PA
UPFD	92.7	95.64	95.64	93	62	95	**99.98**

### Experimental results using variants of the proposed approach

The results of different variants of the proposed approach are generated by replacing the SVM with different classifiers, namely; k-Neighbors, iterative dichotomiser3, multilayer perceptron, AdaBoost, Naive Bayes, and random forest. The different parameter ranges of these classifiers are shown in Table 9. Table 10 shows that the SVM performed well for all datasets (MCF, URFD, UPFD-2P, UPFD-4P) except for three videos in MCF while the second-best performance is shown by kNN, followed by ID3 and random forest classifiers with a very close margin.

**Table 9:** Parameters used for comparison of Decision Tree, Random Forest, KNN, AdaBoost, MLP, and SVM.

Classifier	Parameters
Decision Tree	criterion=‘entropy’, splitter=‘best’, random_state = 0, min_samples_split = 2
Random Forest	criterion=‘entropy’, n_estimators = 10, random_state = 0
KNN	leaf_size = 30, metric=‘minkowski’, n_neighbors = 5, p = 2, weights=‘uniform’
AdaBoost	n_estimators = 50, random_state = None
MLP	activation=‘relu’, alpha = 0.0001, max_fun = 15000, max_iter = 50, random_state = 0, solver=‘adam’
SVM	estimator=‘LinearSV’, max-iter=[500, 1000], C=[0.1,1]

**Table 10:** Comparison of variants of the proposed approach (PA) in terms of accuracy (%) on four datasets namely MCF (Camera 1 to Camera 8), URFD, UPFD (Two Postures), and UPFD (Four Postures).

Datasets	KNN	ID3	MLP	AB	NB	RF	PA
MCF-C1	98.82	95.05	48.52	93.29	78.93	97.71	**99.18**
MCF-C2	98.91	92.88	67.90	83.58	66.15	97.53	**99.20**
MCF-C3	**98.89**	92.85	39.20	91.67	83.41	95.57	98.37
MCF-C4	97.46	96.57	45.04	96.05	94.41	97.46	**97.54**
MCF-C5	**99.41**	97.41	43.95	96.97	94.54	98.23	99.18
MCF-C6	98.90	98.24	69.89	97.43	96.99	98.82	**99.20**
MCF-C7	98.83	97.29	62.20	95.90	95.54	97.95	**98.83**
MCF-C8	**99.26**	96.00	58.28	95.11	85.57	98.07	99.11
URFD	97.38	94.56	58.71	91.31	91.74	96.47	**97.95**
UPFD-2P	100.00	99.94	99.97	100.00	99.73	100.00	**100.00**
UPFD-4P	99.94	99.68	94.47	98.55	98.85	99.74	**99.98**

kNN = k-nearest neighbors, ID3 = Iterative Dichotomiser3, MLP = Multi-layer Perceptron, AB = AdaBoost, NB = Naive Bayes, RF = Random Forest.

### Discussion

We conducted a comparative analysis of the proposed approach against six networks (ResNet-50, ResNet-101, VGG-19, MobileNet, InceptionV3, and Xception) in the context of a four-category classification problem for human posture recognition. The four categories include standing and walking, lying on the ground, moving down or falling, and moving up or jumping. Additionally, we investigated two-category problems (Fall and No Fall) using the UPFD dataset, which comprises videos featuring five distinct types of falls.

The proposed methodology, relying on features extracted from the final pooling layer of ResNet-50 followed by a classification layer incorporating SVM, outperformed existing methodologies and state-of-the-art networks across MCF, URFD, and UPFD datasets, as indicated by the results presented in the previous section. As depicted in Fig 6, our approach exhibited superior accuracy (with a maximum accuracy of 97.95%) compared to ResNet-50, ResNet-101, VGG-19, MobileNet, InceptionV3, and Xception, with VGG-19 displaying the lowest accuracy. The efficacy of our proposed approach is attributed to the optimal margin separability of SVM on robust features derived from ResNet-50. The suboptimal performance of VGG-19 was linked to the *degradation problem*[55], a challenge effectively addressed in the ResNet architecture.

**Fig 8:**
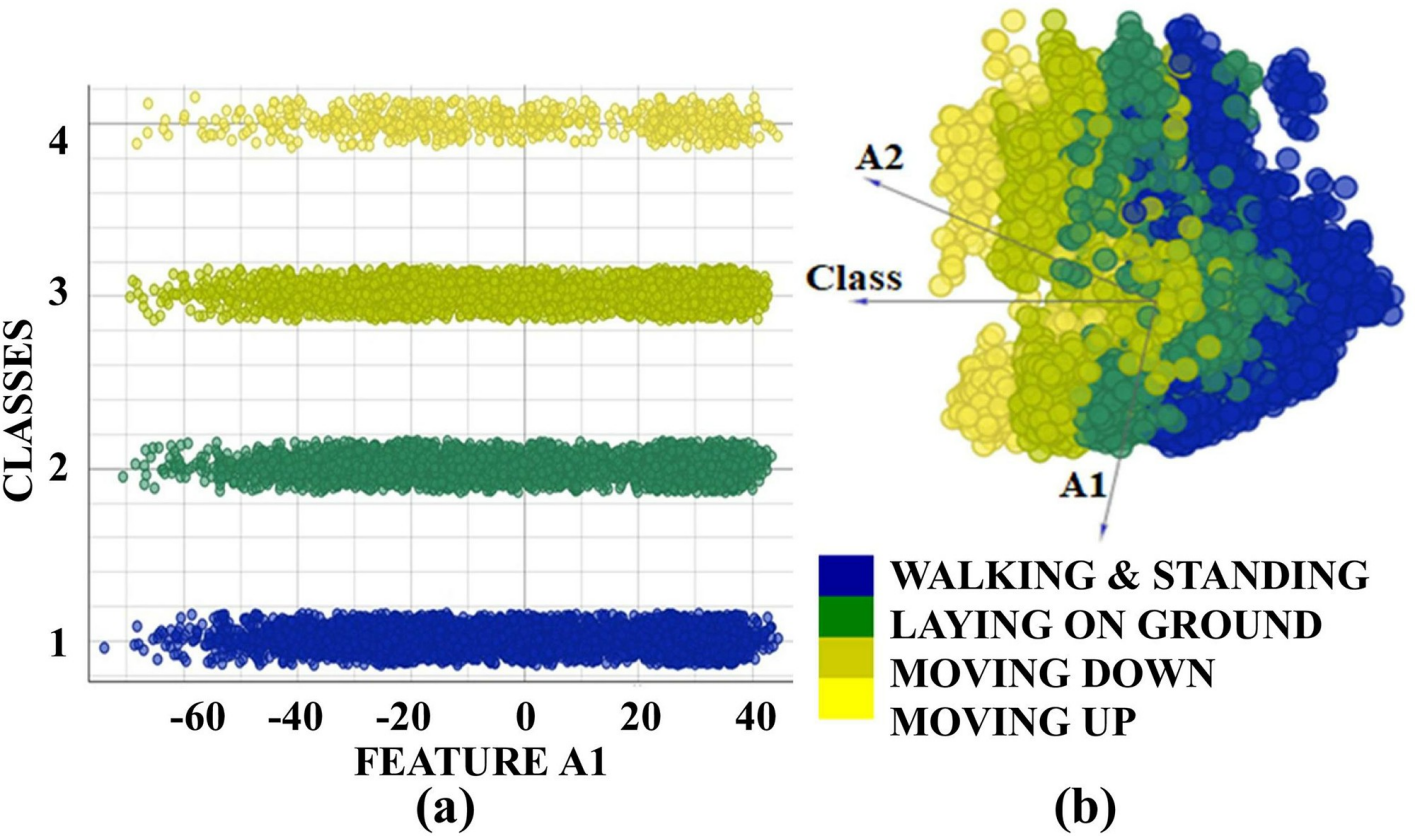
Features (first two principal components (A1 & A2)) of pooling layer of proposed approach of URFD dataset for four human poses including standing or walking, lying on the ground, moving down or falling, and standing up using (a) Scatter plot, (b) Linear projection.

To delve into the feature representation, we conducted principal component analysis (PCA) on ResNet-50 features for the URFD dataset, revealing that the top 119 attributes (out of a total of 2048 features) contained 95% of the information. Fig 8(b) displays the scatter plot for the top two principal components (A1, A2), encapsulating 9% of the information. Conversely, for VGG-19, nearly 90% of the features exhibited negligible magnitudes (i.e., conveyed minimal information). This analysis further strengthens the hypothesis that while considering pre-trained networks, ResNet-50 is a better choice than others since most of its features turn out to be valuable in terms of absolute magnitude when obtained on the human pose classification datasets.

An alternative method to assess the robustness of ResNet-50 features involves comparing the inter-class and intra-class distances of these features. When the inter-class variation exceeds the intra-class variation for the extracted features, it indicates that the features learned by ResNet-50 are semantically suitable for the target labels of the four classes. Table 11 presents the inter-class and intra-class distances for the features extracted from the final pooling layer of ResNet-50 in the URFD dataset. It is evident that the inter-class variation surpasses the intra-class variation for the extracted features; for instance, the inter-class distance between class-3 and class-4 is notably higher than the intra-class distances displayed in the diagonal. The maximum inter-class distance occurs between classes 3 and 4, reaching 3.26, while the minimum inter-class distance is observed for class 1 and class 2, measuring 1.04. However, this minimum distance is far greater than all the intra-class distances. The same can be verified from the classification results obtained for the 4-category classification problem in the URFD dataset in Fig 6. Other two datasets used in our experiments (MCF, UPFD) also exhibited similar behavior. It may be noted that at the pixel level, the training images do not yield this behavior (i.e. higher inter-class variation than the intra-class variation is not observed for the training or validation datasets). Eqs (3) and (4) were used to calculate intra-class and inter-class distances.

$$\begin{array}{c} S_{a}=\frac{\sum_{i,i^{\prime }}∥x_{i}-x_{i^{\prime }}∥}{N_{k}\left(N_{k}-1\right)} \end{array}$$ (3)

Here, *x*_*i*_ is the number of intra-class feature attributes and *N*_*k*_ is the total number of vectors.

$$\begin{array}{c} d_{a}=\frac{\sum_{i,j}∥x_{i}-x_{j}∥}{N_{k}N_{i}} \end{array}$$ (4)

*x*_*i*_ − *x*_*j*_ is the distance of inter-class vectors, *N*_*k*_*N*_*i*_ shows total number of vectors.

**Table 11:** Within-class (average distance) and between-class(average linkage) distances for URFD dataset (ResNet-50 features).

Class	Class1	Class2	Class3	Class4
Class1	0.66	1.04	1.11	2.47
Class2	1.04	0.96	1.44	1.47
Class3	1.11	1.44	0.70	3.26
Class4	2.47	1.47	3.26	0.79

**Fig 9:**
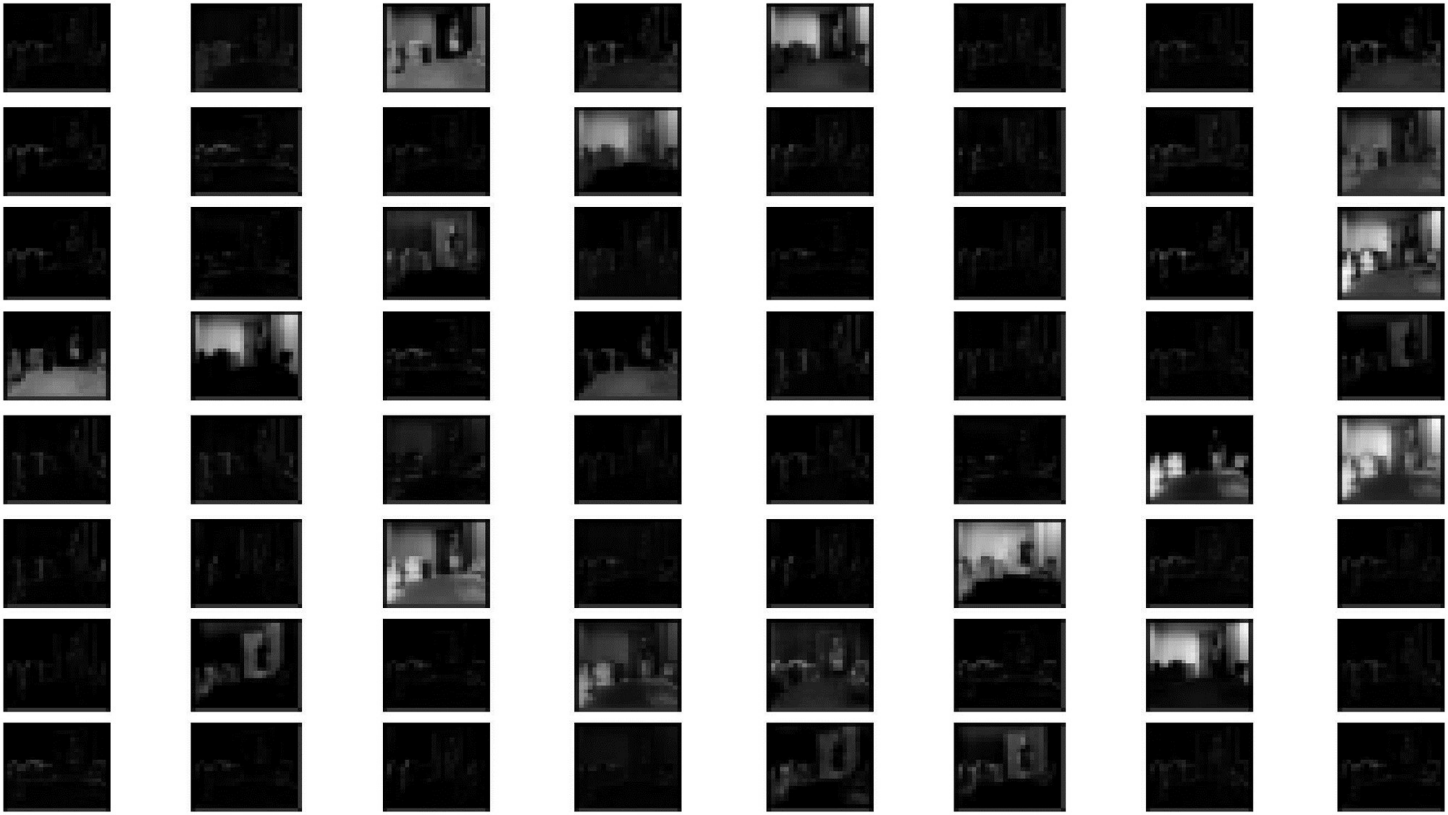
VGG-19 low-level feature visualization of URFD dataset for human pose standing or walking.

**Fig 10:**
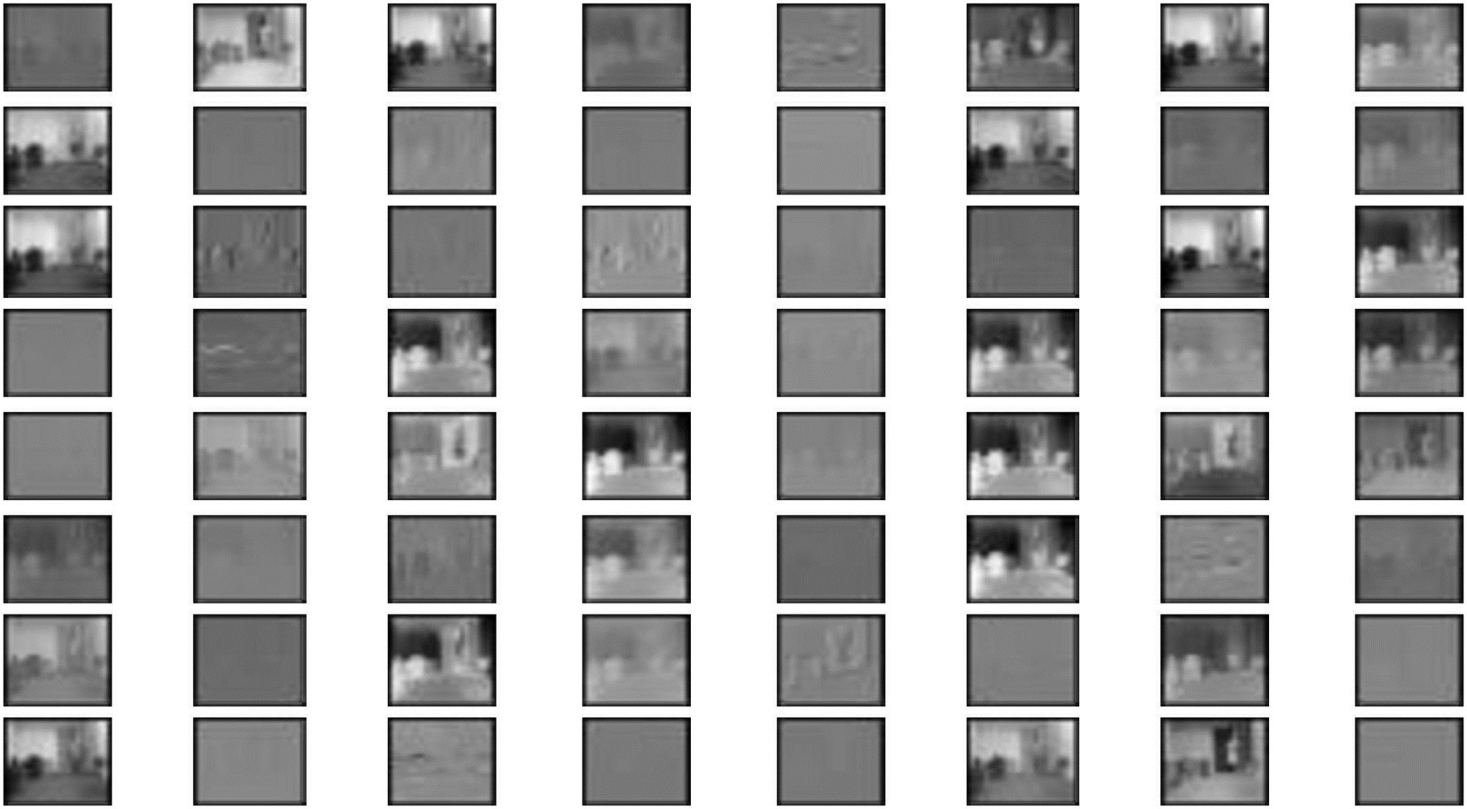
ResNet-50 low-level feature visualization of URFD dataset for human pose standing or walking.

We also analyzed activation maps generated after applying the learned filters to the input, at different convolutional layers. Fig 9 shows activation maps from the first layer (i.e. low-level features) of VGG-19 network and Fig 10 shows the same for the ResNet-50, for a posture of standing and walking from the URFD dataset. The low-level features in the ResNet-50 (variation of gray-scale images) are better representative of image pattern dynamics than those of the VGG-19 which are mostly dark patterns. Similar quality of feature discrimination was also exhibited by the remaining layers of ResNet-50 for the other categories of the dataset.

## Conclusion and future works

This work focused on posture recognition for elderly people. We combined the deep features of the residual network with SVM to outperform state-of-the-art approaches both in terms of accuracy and time efficiency on MCF, URFD, and UPFD datasets. Moreover, the SVM outperformed k-nearest neighbors, iterative dichotomiser3, multilayer perceptron, AdaBoost, Naive Bayes, and random forest as the classification layer. In experimental results, our framework proved highly robust against varying spatial locations, varying lighting conditions, scale changes, camera noise, and different viewpoints. The proposed approach outperformed other approaches on the MCF dataset, achieving an accuracy of 98.82%, while the second-best accuracy (97.9%) was obtained by Fan. Similarly, our proposed approach outperformed other approaches on the URFD and UPFD datasets, achieving an accuracy of 97.95%, and 99.98% respectively. The second-best accuracies (97.33%, 95.65%) on the aforementioned datasets were obtained by Wang and Espinosa respectively. Our hybrid approach can be deployed in old people’s care centers and hospitals to monitor posture and provide first aid in the event of injury.

In the future, we will explore human posture recognition in a more challenging environment such as poorly lit areas and crowds. We also plan to explore our method for thermal images, e.g. for elderly people at risk of falling in dark rooms. Depending on how complex the environment of everyday life is, surveillance may not be able to completely capture people’s actions. People’s behavior and actions can be estimated and predicted in the presence of partial occlusions. When a fall occurs, the rescuer should be notified promptly of the scene, location, time, and other details to enhance the speed of emergency rescue. We plan to evaluate the performance of the proposed approach on new datasets and also investigate the impact of combining these datasets. This approach will help us assess and enhance the model’s robustness and accuracy. Moreover, we plan to discuss potential interference factors such as patient variability (age, body composition), environmental conditions (lighting, obstacles), and sensor placement inconsistency, which may impact our model’s performance in clinical scenarios. Additionally, we plan to evaluate the effectiveness of models (EfficientNet, CoAtNet) on the task of human posture using accuracy, time efficiency, precision, recall, and p-test. The EfficientNet model scales the properties of MobileNets and ResNet, whereas the CoAtNet model provides good generalization like ConvNets and superior model capacity like Transformers.
